# Marketable value estimation of patents using ensemble learning methodology: Focusing on U.S. patents for the electricity sector

**DOI:** 10.1371/journal.pone.0257086

**Published:** 2021-09-13

**Authors:** Haneul Eom, Sungyun Choi, Sang Ok Choi

**Affiliations:** 1 Program in Science & Technology Studies, Korea University, Seoul, South Korea; 2 School of Electrical Engineering, Korea University, Seoul, South Korea; 3 Department of Public Administration, Korea University, Seoul, South Korea; Vietnam National University, VIET NAM

## Abstract

Patent valuation is required to revitalize patent transactions, but calculating a reasonable value that consumers and suppliers could satisfy is difficult. When machine learning is used, a quantitative evaluation based on a large volume of data is possible, and evaluation can be conducted quickly and inexpensively, contributing to the activation of patent transactions. However, due to patent characteristics, securing the necessary training data is challenging because most patents are traded privately to prevent technical information leaks. In this study, the derived marketable value of a patent through event study is used for patent value evaluation, matching it with the semantic information from the patent calculated using latent Dirichlet allocation (LDA)-based topic modeling. In addition, an ensemble learning methodology that combines the predicted values of multiple predictive models was used to determine the prediction stability. Base learners with high predictive power for each fold were different, but the ensemble model that was trained on the base learners’ predicted values exceeded the predictive power of the individual models. The Wilcoxon rank-sum test indicated that the superiority of the accuracy of the ensemble model was statistically significant at the 95% significance level.

## Introduction

With the advancement of the industrial infrastructure, such as Industry 4.0, the importance of intangible assets continues to increase, and patents, a representative intangible asset, are considered a key resource for enhancing a corporation’s technological competitiveness [1]. Despite the importance of patents, patent transactions in the market are not active due to difficulties in determining a reasonable value that consumers and suppliers can satisfy [2].

Traditionally, patent valuation methodologies have been largely divided into revenue, cost, and market approaches [3]. The revenue approach is a way to convert future revenue from patent rights to the present value. Because the revenue approach is based on predictions of uncertain futures, it has been difficult to calculate prices that both consumers and suppliers can understand. In contrast, the cost approach has advantages in that it is easy to measure and requires less subjective factors. However, the cost approach has the inherent limitation of not considering the profitability or market value of the patent. The market approach determines the value based on the market-traded price, and the proper value can be determined because overvalued or undervalued patents converge at an appropriate price through repeated transactions. However, unlike the stock and raw material markets, the patent market has been difficult to value through a market approach because patents are less frequently traded [4].

This study complements the limitations of the market approach using machine learning and an event study methodology. Although research using machine learning for patent analysis has been actively conducted [5–8], most of this research is limited to technology classification, technology prediction, and patent quality analysis. Few studies have used machine learning for patent valuation because securing patent transaction data to train a machine learning model is difficult, as most patents are privately traded to prevent technical information leaks [9].

In this study, the event study methodology is used to overcome these limitations. An event study can be applied to extract the judgment of market participants on the patent by controlling the common market factors from the stock rate at the patent enrollment date. Daily stock and index returns are collected from a certain point in time to the patent enrollment date to employ an event study, and a regression analysis is conducted on these returns. Based on the regression analysis results, the expected normal return is calculated. The abnormal return is also calculated by subtracting the expected normal return from the stock return at the patent enrollment date. In this study, the marketability value of patents is represented based on abnormal returns.

Topic modeling was performed using latent Dirichlet to analyze the semantic information of the patent text. The computed abnormal return was then combined with the topic information of the patent produced using topic modeling as training data for the machine learning-based ensemble learning model. Through this, the market value of a patent can be estimated using the machine learning methodology.

In this study, the ensemble method was used to secure a stable predictive power. A single model has an inevitable model bias problem. An ensemble model that calculates the final predicted value based on the predicted values of several models can reduce the model bias.

## Related work

Related research can be divided into two parts: patent valuation and patent topic modeling.

### Patent valuation

Patent valuation studies have been conducted based on cost [10–12], revenue [13–15], and market approaches [16], with market approaches centered on small-scale cases where patent transaction information exists. This study is a complementary approach that bypasses market-approach limitations because it uses the event study methodology to extract the marketable value of patents. An event study is a methodology to study the influence of a particular event on stock prices [17–19].

Three typical methods of estimating the normal return are the mean adjusted return method, market adjusted return method, and market model [20]. The mean adjusted return method estimates the mean return during the period before the event as a normal return, and the market adjusted return method estimates the normal return using the market rate of return during the event period [21]. In contrast, the method using the market model calculates the normal return during the event period using the result of estimating the market return sensitivity $\left(\hat{\alpha },\;\hat{\beta }\right)$ of the stock return using the ordinary least square (OLS) regression analysis [22]. Research using event studies for technology valuation has been actively conducted. Studies have evaluated the value of innovation through event research [23–25]. Research using event studies for patent valuation has also been actively conducted [19, 26, 27]. In this study, the marketable value of the patent is represented by the abnormal return, and the abnormal return of the patent is matched with the topic information produced through topic modeling.

### Patent topic modeling

Research on topic modeling has actively been studied. Topic modeling is divided into non-probabilistic and probabilistic models [28]. The probabilistic approach yields a probability distribution over a set of classes for each input sample. In contrast, non-probabilistic models separate the functional space without modeling the class distribution and return the classes associated with the space from which the sample originated.

A representative non-probabilistic approach is to cluster keywords belonging to patent documents using the K-means method [29]. Research using a patent ontology network for extracting the K-nearest neighbors [30] or using the fuzzy set methodology for subject evaluation [31] has also been conducted. However, non-probabilistic models have limitations in seizing the semantic effect of patent content.

The probabilistic model is effective in discovering the hidden subject structure of documents. Various LDA-based models have been widely adopted depending on various aspects of patent data. Some research has combined patent information with an LDA-based subject model to discover potential semantic subjects [32] and has explored patent-related segment topics using the document structure [33].

In this study, we conduct topic modeling of patent text information using the LDA, a probabilistic methodology. We use machine learning-based ensemble models to evaluate the marketable value of patents based on their textual information. Although related studies that directly estimate the market value of a patent using machine learning techniques have been insufficient, some studies have predicted patent quality using patent citations as a dependent variable [34–37]. Various machine learning algorithms predict dependent variables, such as patent quality, using patent text information, such as neural network-based models [38], support vector machine (SVM)-based models [39], and random forest (RF)-based models [40].

A single model may cause a deflection problem, whereas the ensemble technique potentially reduces deflection. We could not find any studies where machine learning and ensemble techniques were used in patent valuation because patent trade price information is not disclosed. However, AdaBoost, in which a decision tree is set as a base learner, was used as an ensemble in technical transfer prediction, which is a different field [41]. Furthermore, in the entity recognition of patents, a voting-based ensemble in which CRF, CNN, and BERT were set as base learners was used [42]. More recently, a methodology to measure an internal quality measure through a graph-based unsupervised ensemble has been attempted [43]. The present study combines event study methodology and topic modeling and calculates the final prediction value through an ensemble. Specifically, various base learners are used, while support vector regression (SVR) is used as a meta learner in order to account for the deflection of the base learner when calculating the final prediction value. Table 1 presents the differences between related work and the model proposed in this study.

**Table 1:** Comparison with the related studies.

Division		Related work				Proposed model
Patent valueation	Input variable	[19, 23–27]	Stock price			Patent text information, Stock price
	Methodology		Event study			Topic modeling, Event study
Ensemble learning	Forecast target	[41]	Technology transfer	[42]	Named entity recognition	Patent valuation
	Base learner		Decision tree		CRF, CNN, bert	Random forest, MLP, CNN
	Meta learner		Ada-boost		Voting	Stacking Ensemble (SVR)
	How to derive a target variable	[43]	Unsupervised learning (calculation of internal quality measure)			Event study (calculation of patent market value)

## Methodology

### Proposed methodology and framework

**Fig 1:**
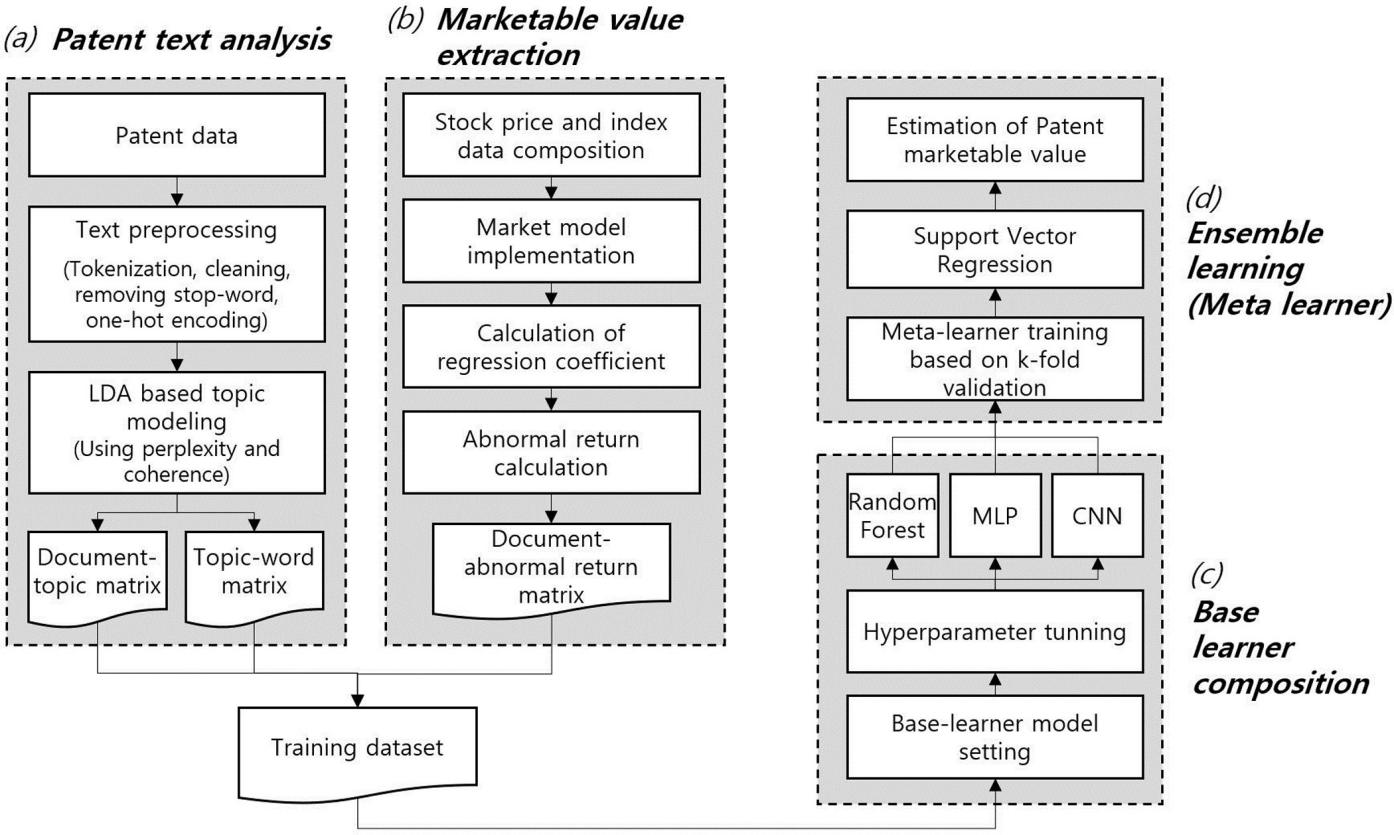
Framework for marketable value estimation of patents. [a: The topic allocation probability used as an independent variable of the base-learner is calculated through LDA-based topic modeling. b: The market value of a patent used as a dependent variable is calculated through the event study methodology. c: Random Forest, MLP, and CNN are used as base learners. d: SVR is used as the algorithm of the ensemble model that calculates the final predicted value through the predicted value of the base-learner].

This work combines the marketable values of patents produced through an event study with the topic information produced by topic modeling. The corresponding data are used for training machine learning-based ensemble models. This training allows the ensemble model to estimate the marketable value of the patent when given an arbitrary patent. The patent data used for patent valuation are U.S. registered patents for the electricity (IPCH) sector from April 1999 to June 2020. An analysis was conducted only on patents held by listed companies with stock price information to link to the event study. The abstract information of the patents was converted to a corpus, and an LDA analysis was performed. Afterward, the optimal number of topics was calculated using perplexity and coherence analyses, and topic modeling was performed based on the optimal number of topics. To determine the marketable value of a patent, the past 30 daily stock price returns and index returns, including the date of patent enrollment, were collected. A regression equation was constructed using the calculated stock price and index return to calculate the abnormal return. In this study, the abnormal return was used as the market value of the patent. The base learner learns based on the calculated market value of the patents and patent topic information. As a base learner, the RF, multilayer perceptron (MLP), and convolutional neural network (CNN) were used. The meta learner learns using the predicted value calculated using each base learner and estimates the market value of the patent. In this study, SVR was used as a meta learner. Fig 1 presents the framework for marketable value estimation of patents.

### Patent text analysis

Topic modeling is one of the text mining techniques for extracting meaningful topics from unstructured text data. It operates on the principle of deriving and classifying the topics embedded in a document based on the words that comprise the document in a large corpus [44]. Representative models of topic modeling are probabilistic latent semantic indexing (pSLI) [45], and LDA models have improved on pSLI [46, 47].

The LDA model is an unsupervised learning method that finds hidden topics within a document through learning that consists only of word patterns, the only observed data, without knowing what each document contains. This method is also called a probabilistic generative model for a set of documents.

The LDA assumes that documents have multiple topics, and each subject follows a Dirichlet distribution. When M documents are given and all documents belong to one of K topics, the word can be represented as an index of the vocabulary. If the size of the vocabulary is V, each word corresponds to an index *v* = {1,…,*V*}. The word vector w is expressed as vector V and satisfies *w*^*v*^ = 1, *w*^*u*^ = 0, *v* ≠ *u*. In other words, if the document contains a v word, it is marked as 1; otherwise, it is marked as 0. Document W is written as *W* = (*w*_1_,*w*_2_,…,*w*_*N*_) in N consecutive words. The corpus is a set of documents, expressed as *D* = {*W*_1_, *W*_2_, …, *W*_*N*_}.

**Fig 2:**
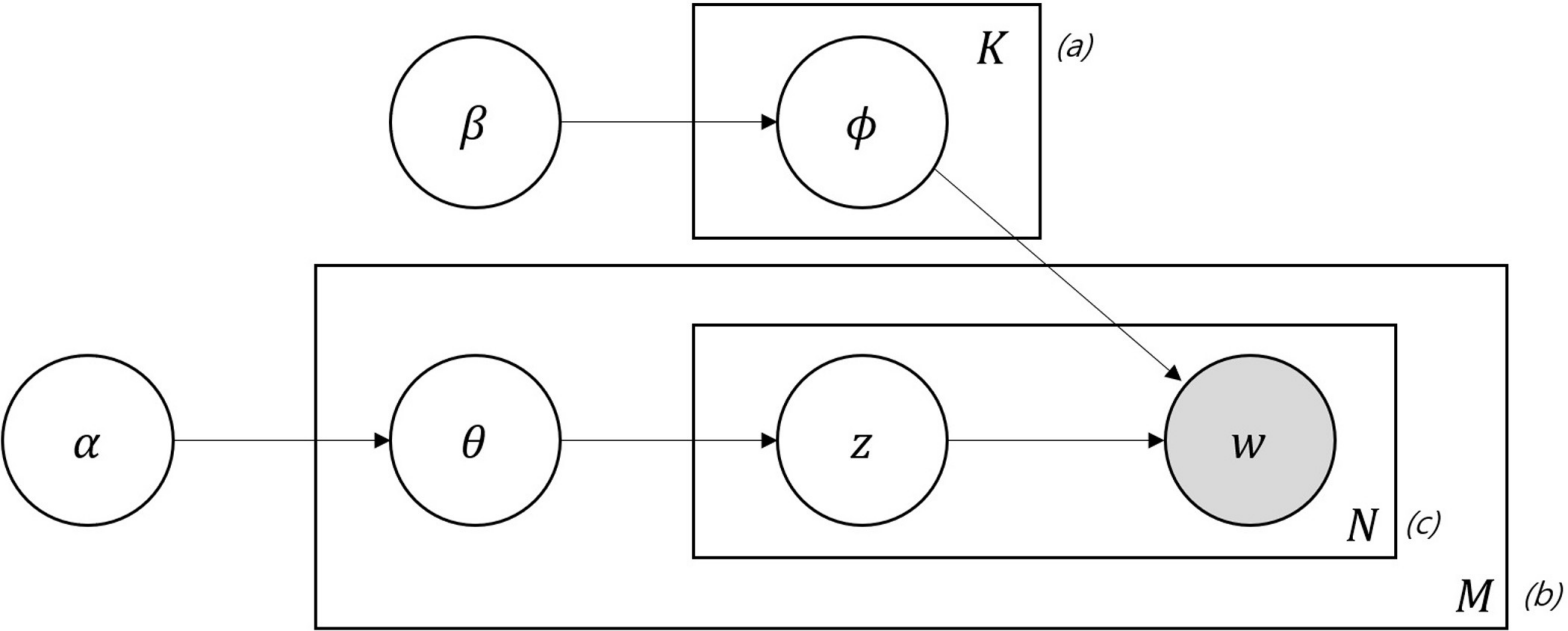
Graphical model representation of the LDA model. [a: K stands for the number of topics. b: M indicates the number of documents. c: N represents the total number of words in the document].

Fig 2 illustrates a probability graph model for this, where K stands for the number of topics and assumes a fixed, known value, M indicates the number of documents, and N represents the total number of words in the document. In addition, *α* is a hyperparameter with the same value in the set of documents. Moreover, *α* is a parameter of the K-dimensional Dirichlet distribution and determines *θ*, which represents the proportion of subjects in each document and is a K-dimensional vector. Further, *θ*_*i*_ is the probability that the document belongs to the *i*th subject. That is, it indicates the distribution of the subject of the document and satisfies $\Sigma_{i=1}^{K}\;\theta_{i}=1$. Additionally, *Z* is an N-dimensional vector, and *z*_*n*_ is a subject assigned to the word w. In the figure, β is a matrix of size *K* × *V*, and *β*_*ij*_ is the probability that the *i*th subject generates the *j*th word of the vocabulary. Here, *w*_*n*_ is given through actual documentation, and the other variables are latent variables that cannot be observed. Moreover, *β* is a parameter of a Dirichlet distribution and determines ϕ. Here, *ϕ* indicates the proportion of the subject each document comprises. In addition, *ϕ* is a K-dimensional vector, and *ϕ*_*i*_ is the probability that the document belongs to the *i*th subject. In other words, it is the distribution of the subject of the document and satisfies $\Sigma_{i=1}^{K}\;\phi_{i}=1$. To use the LDA, given a document, a posterior distribution must be derived for the latent variable Z.

The topic model combines words belonging to the same semantic class into one subject based on the simultaneous occurrence of words in a document set. This process does not require manual labor; thus, large documents can be processed without a problem. However, as with unsupervised learning, it is not known how much the automatically processed results match the desired results, which is the most important limitation of the unsupervised topic modeling method.

Therefore, the task of evaluating the performance of the topic modeling results is emerging as important. Two categories of methods exist to solve this problem. Unlike intrinsic methods that do not use external sources of the dataset, external methods use topics discovered by external work, such as information retrieval or external statistics, to evaluate topics.

In this study, perplexity [48], a classical technique for implicitly evaluating a topic model, and the coherence technique [49], which complements the limitations of the perplexity analysis, were used together. Perplexity is an index that measures how well the probability model predicts a sample in information theory, and a lower perplexity value indicates better predictive power. Perplexity is primarily used to evaluate how well the probability model improves compared to other models or to evaluate performance according to parameters within the same model:

$$Perplexity=exp\left[-\sum_{d=1}^{M}\log\;p(\omega_{d})/\sum_{d=1}^{M}\omega_{d}\right]$$ (1)

where *p*(*ω*_*d*_) denotes the probability that a specific word in the *d*th document is assigned to the subject, and *N*_*d*_ represents the total number of words in the dth document. A low perplexity value indicates that learning is well done, not that the result is good for human interpretation. Low perplexity values do not always exhibit adequate results for interpretation [50].

The coherence analysis complements this aspect. Coherence is an index that measures the semantical consistency of a topic. More consistent words indicate a higher coherence. Coherence is primarily used to determine the meaningfulness of the information produced by the model. The measurement index of coherence used in this study is as follows:

$$Coherence=log\;\frac{p(\omega_{i},\omega_{j})}{p(\omega_{i})p(\omega_{j})},\;i\neq j$$ (2)

where *p*(*ω*_*i*_, *ω*_*j*_) represents the probability that the *i*th and *j*th words appear simultaneously in each document, and *p*(*ω*_*i*_) denotes the probability that the *i*th word appears in the entire document set.

The Gibbs algorithm is a method of inferring that only one variable is changed while the remaining variables are fixed, and unnecessary variables are excluded. Numerous calculations are required to determine the posterior distribution considering all latent variables; thus, only the posterior distribution for the topic Z of interest was considered. The posterior distribution for *Z* is as follows:

$$p(Z|W,\alpha ,\beta )=\int \int \prod_{i=1}^{K}p(\phi_{i}|\beta )\prod_{i=1}^{K}p(\theta_{d}|\alpha )\prod_{i=1}^{K}p(z_{dn}|\theta_{d})\;p(w_{dn}|\phi_{z_{dn}})d\phi d\theta$$ (3)

For sampling in the Gibbs algorithm, a conditional distribution must be defined, and the posterior distribution for Z can be defined from the relationship between the Dirichlet distribution and the multinomial distribution in the LDA probability model:

$$p(z_{n}=k{|z}_{-n},W)\approx \frac{n_{k,-n}^{\left(v\right)}+\beta_{v}}{\sum_{v=1}^{V}n_{k,-n}^{\left(v\right)}+\beta_{v}}\times \frac{n_{d,-n}^{\left(k\right)}+\alpha_{k}}{\sum_{k=1}^{K}n_{d,-n}^{\left(k\right)}+\alpha_{k}}$$ (4)

where *α* and *β* are parameters of the Dirichlet distribution. In addition, $n_{k,-n}^{\left(v\right)}$ refers to the frequency at which the *v*th word is observed as the kth topic among the remaining words excluding the nth word *w*_*n*_ in each document, and $n_{d,-n}^{\left(k\right)}$ refers to the frequency of words with subject k in the document, excluding the nth word in document *d*. A topic is randomly assigned to all words in the document set, and the topic distribution for each document to which the word belongs and the word distribution for each topic are calculated except for the nth word. Sampling is performed using the above equation, and the process is repeated until convergence.

### Marketable value extraction

An event study is a methodology that measures the effect of unexpected events or the disclosure of new corporation information on the expected profitability of a corporation. The event study is used to extract market value in this study. A decision or situation that is considered to have influenced the stock price is called an event. The day when the stock price is considered to have been affected by the event is called an event day. A normal return is the rate of return calculated based on the normal stock price that would have been formed if the event had not occurred. Three typical methods of estimating a normal return are the mean adjusted return method, market adjusted return method, and market model. The mean adjusted return method estimates the mean return during the period before the event as a normal return, and the market adjusted return method estimates the normal return using the market rate of return during the event period. In contrast, the method using the market model calculates the normal return during the event period using the result of estimating the market return sensitivity $\left(\hat{\alpha },\hat{\beta }\right)$ of the stock return using the OLS regression analysis.

In the market model, the normal return of the event period is calculated by substituting the explanatory variable data during the event period into the regression model that estimates the normal return. The target period for extracting the necessary data to measure the parameters of the regression model is called the estimation period. In this study, the normal return is calculated through OLS based on the stock price and index returns over a certain past period from the patent registration date. At time *t* when patent *i* is registered, the stock price return *R*_*i*,*t*_ of the corporation holding the patent can be expressed as follows according to the market model:

$$R_{i,t}=\alpha_{i}+\beta_{i}\times RM_{t}+e_{i,t}$$ (5)

where *RM*_*t*_ denotes the total market return at time t, *e*_*i*,*t*_ represents the intrinsic rate of return of the corporation holding patent *i* at time *t*, which is not correlated with the entire market and is assumed to have an expected value of 0. In addition, *β* is the sensitivity of the analysis target stock return to the market return, and *α* is the expected return of the stock when the market return is zero.

Therefore, Eq (5) can be explained as dividing the stock price return (*R*_*i*,*t*_) of the analyzed stock into the rate of return by market factors and the rate of return by factors specific to the stock. The explanatory variable used in the market model is the rate of return of the index representing the entire market. In this study, Standard and Poor’s 500 (S&P 500) is used as a representative index. The abnormal return is calculated by subtracting the normal daily return estimated by the model from the actual rate of return during the event period. The abnormal rate of return calculated in this way is also referred to as an excess return in the sense that the rate of return exceeds the normal return by the market model. The rate of return *AR*_*t*_, which is changed according to the occurrence of a patent event, can be expressed as follows using the above equation:

$$AR_{i,t}=R_{i,t}-E(R_{t}|RM_{t})=R_{t}-\alpha_{i}-\beta_{i}\times RM_{t}$$ (6)

where *E*(*R*_*i*,*t*_ | *RM*_*t*_) is the expected return of the corporation holding the patent given the market return *RM*_*t*_.

### Base leaner composition

#### Random forest model

The RF model is a method of learning using multiple decision trees. A forest comprises uncorrelated trees using a classification and regression tree (CART), such as a method that combines random node optimization and bagging. The CART method is a technique to determine the effect on the response variable (dependent variable) by making the most of the nonlinearity and interactions of the explanatory variable or predictor. Moreover, the CART branches of explanatory variables are created according to the importance criterion, and they make judgments on response variables at the last node. The CART can be used even when the response variable is binomial, polynomial, or continuous. A predictor (explanatory variable) can also be selected without distinction between continuous and categorical variables. Like CART, RF can be used for both categorical and continuous response variables. The same algorithm as the CART is used to build the tree.

However, for the CART method, one decision tree is derived, whereas forming a forest comprising numerous decision trees is different in RF. The random sampling of predictors and observations is repeated to build multiple decision trees. After obtaining a categorical prediction from numerous decision trees, the final categorical prediction is decided by majority voting. By providing randomness to decision tree formation, independent decision trees can be repeatedly created, and prediction errors can be reduced. The bootstrapping technique is used for the random selection of predictors and observations. For the CART method, as the number of lower nodes increases, the bias of the prediction error decreases, but the variance increases. However, in the RF method, the variance of prediction errors can be reduced by repeatedly generating equally distributed decision trees.

#### Multilayer perception model

The multilayer perceptron is a neural network model consisting of an input layer, a hidden layer, and an output layer. In artificial neural networks, a methodology for configuring and analyzing multiple hidden layers is called the MLP. This MLP model is an analysis methodology that is widely used in pattern classification, recognition, and prediction and is extended to more advanced artificial neural network analyses according to the shape and activity function of the hidden layer. This is a more advanced algorithm than the single-layer perceptron, consisting of one basic input layer and a hidden layer. It could be expressed in the form of Eq (7):

$$Y_{k}=\sum_{j=1}^{m}f\;\left(\sum_{i=1}^{n}v_{i}w_{ij}+b_{j}\right)\;w_{jk}+b_{k},$$ (7)

where *v*_*i*_ is a signal of the input layer or the previous hidden layer, and *b*_*j*_ and *b*_*k*_ indicate the bias between the hidden and output layers, respectively. In addition, *w*_*ij*_ and *w*_*jk*_ denote the coefficient values of the hidden and output layers, respectively. Further, *f* represents the activation function, and the sigmoid and ReLU functions are commonly used. The resulting value (*Y*_*k*_) of the output layer can be obtained through Eq (7).

#### Convolutional neural network model

The CNN exhibits remarkable performance in the field of image identification. This methodology makes a value that recognizes only a certain part of the image into a new value through a filter. The CNN connects these values continuously so that the image characteristics can be better understood. The CNN uses the convolution of an input layer and filter and comprises one input and one output layer, one or more convolutional layers, and a pooling layer. The data are input through the input layer and filtered through the convolutional layer to extract the appropriate features. The number of feature maps is determined according to the number of filters.

### Ensemble learning

No single model can perform well in all situations. The stacking ensemble model used in this study creates a model with the best performance by combining different models. The stacking ensemble model can be configured by combining various algorithms. Through these combinations, weaknesses can be compensated for while taking advantage of each algorithm. Thus, the performance can be improved over a single model that is generally trained. In this study, a stacking ensemble model is constructed using the RF, MLP, and CNN models.

To calculate the predicted value for each submodel to be used as the input data for the stacking ensemble model, the training set was again divided into seven subtraining data, and the submodel training and predicted value calculations were performed for each fold. As an algorithm for ensemble learning, we used the SVR, which has better interpretation power than the black-box model. The SVR is a generalization technique of the SVM, a classification algorithm [51], and predicts data by determining an optimal hyperplane that includes as much data as possible within the distance between support vectors.

### Model comparison

Table 2 lists the main logic and data structures of the RF, MLP, CNN, and stacking ensemble models used to predict the marketable value of a patent. Models 1 to 3 are the experimental group for comparing the predictive power of the stacking ensemble model. The base learners used in Model 4 are the same as the logic of Models 1 to 3 but were trained and tested models in a set of seven divided training data (seven-fold). The meta learner was trained based on the base learners’ predictions calculated from the testing set of each fold. The MLP was used as an algorithm that combines the predicted values of the base learners.

**Table 2:** Model comparison.

Division	Model 1	Model 2	Model 3	Model 4
	Random Forest	MLP	CNN	Stacking Ensemble
Main logic	Decision tree	Neural network	Neural network	Combining predictions using SVR
Training input data	Training set	Training set	Training set	Base learner (RF, MLP, CNN) prediction results using segmented training set (seven-fold)
Testing input data	Testing set	Testing set	Testing set	Base learner prediction results in the testing set
Training data dimensions	(19226, 80)	(19226, 80)	(19226, 80)	(19226, 3)
Testing data dimensions	(8239, 80)	(8239, 80)	(8239, 80)	(8239, 3)

The mean absolute error (MAE), mean squared error (MSE), and root mean squared error (RMSE) are used as indicators to evaluate the model’s performance and calculated as follows:

$$\text{MAE}=\frac{1}{\text{n}}\sum_{i=1}^{n}\left|e_{t}\right|$$ (8)

$$\text{MSE}=\frac{1}{\text{n}}\sum_{i=1}^{n}e_{t}^{2}$$ (9)

$$\text{RMSE}=\frac{1}{\text{n}}\sum_{i=1}^{n}\sqrt{\frac{1}{n}\sum_{t=1}^{n}e_{t}^{2}}$$ (10)

Next, to determine whether a statistically significant difference exists between the stacking ensemble model and predicted values of each individual model, a pair consisting of the stacking ensemble model and each individual model is constructed. The Anderson–Darling normality test is performed to determine whether the pair had normality. The Anderson–Darling test statistic is defined as follows:

$$A^{2}=-N-S$$ (11)

$$\text{where}\;S=\Sigma_{i=1}^{N}\frac{\left(w_{i}-1\right)}{N}[\text{ln}\;F(Y_{i})+\text{ln}(1-F(Y_{N+1-i}))].$$

The Wilcoxon rank-sum test is a nonparametric alternative to the two-sample t-test which is based solely on the order in which the observations from the two samples fall. The test statistic was computed as follows: *W* = min(*w*_1_, *w*_2_), where W1 and W2 are the sums of the combined rank from Groups 1 and 2, respectively. For *n*_1_ and *n*_2_ > 10, the normal approximation can used (i.e., *W* is normally distributed with mean *μ*_*w*_ = *n*_1_(*n*_1_ + *n*_2_ + 1)/2 and standard deviation $\sigma_{w}=\sqrt{n_{1}n_{2}(n_{1}+n_{2}+1)/12)}$. The Z-statistic can be computed as follows:

$$Z_{0}=\frac{W-\mu_{w}}{\sigma_{w}}$$ (12)

where *Z*_0_ is approximately normal with a mean of 0 and a variance of 1.

## Experiment setup

### Data description

In this study, among the electricity sector (IPC H) patents registered with the U.S. Patent Office from April 1999 to June 2020, the abstracts of the patents held by listed companies with stock price information were used as the text analysis data. The data were preprocessed to analyze patent text information, which is unstructured data. For example, converting words to lowercase, tokenizing a sentence by word, removing stop words to remove articles causing noise in the analysis, and stemming were conducted. The stock return and S&P 500 rate data from the past 30 days at the date of patent enrollment were used as basic data for determining the marketability of patents.

### Patent text analysis

A total of 27,464 patent documents were used for analysis, and the abstract information from each patent was used for text analysis. The average number of words in each document was 67, and the maximum and minimum word counts were 3,576 and 5, respectively. The standard deviation of the number of words by document was 49.

The words and their weights for each topic are listed in Table 2. The topic relevance of each document is presented in Table 3. The rows of Table 4 indicate the patent document number, and the columns indicate the dominant topic for each document and the probability of being included in the individual topics. The probability values in the topic were matched with the marketable value of the patent and used for ensemble leaner learning. The mean and standard deviation were 0.012285 and 0.037705, respectively.

**Table 3:** Words and their weights for each topic.

Topic (Sample)	Expression
Topic 0	’0.132* “include” + 0.124* “embodiments” + 0.072* “methods” + 0.065* “memory” + 0.051* “herein” + 0.050* “described” + 0.046* “disclosure” + 0.037* “disclosed” + 0.033* “aspects” + 0.030* “various”’),
Topic 1	’0.139* “semiconductor” + 0.119* “substrate” + 0.065* “forming” + 0.057* “formed” + 0.042* “method” + 0.031* “includes” + 0.030* “mask” + 0.030* “material” + 0.023* “doped” + 0.022* “device”’),
Topic 2	’0.092* “frame” + 0.074* “block” + 0.052* “resources” + 0.035* “motion” + 0.034* “frames” + 0.028* “subset” + 0.028* “coding” + 0.028* “blocks” + 0.026* “based” + 0.023* “decoding”’),
Topic 3	’0.159* “dielectric” + 0.157* “material” + 0.097* “electrode” + 0.061* “stack” + 0.039* “forming” + 0.024* “electrodes” + 0.024* “bottom” + 0.022* “activity” + 0.022* “spacer” + 0.021* “sidewall”’),
Topic 4	’0.183* “line” + 0.144* “field” + 0.082* “lines” + 0.069* “uplink” + 0.057* “downlink” + 0.056* “pair” + 0.029* “single” + 0.024* “arrays” + 0.022* “waveform” + 0.020* “continuous”’),
Topic 5	’0.076* “wall” + 0.066* “vehicle” + 0.059* “slot” + 0.056* “window” + 0.052* “usage” + 0.049* “card” + 0.047* “analysis” + 0.039* “interior” + 0.036* “dual” + 0.033* “outside”’),
⋮	⋮
Topic 79	’0.383* “plurality” + 0.143* “node” + 0.061* “respective” + 0.056* “nodes” + 0.030* “inductor” + 0.022* “intermediate” + 0.022* “common” + 0.017* “wherein” + 0.015* “configured” + 0.014* “comprises”’)]

**Table 4:** Dominant topics for each document and the probability of being included in individual topics.

Doc.	Dominant Topic	Topic 0	Topic 1	Topic 2	Topic 3	Topic 4	Topic 5	⋯	Topic 78	Topic 79
0	20	0.000000	0.000000	0.000000	0.000000	0.000000	0.000000	⋯	0.000000	0.000000
1	65	0.000000	0.047211	0.000000	0.000000	0.000000	0.032653	⋯	0.000000	0.000000
2	65	0.000000	0.066626	0.000000	0.073362	0.000000	0.041079	⋯	0.026154	0.000000
3	38	0.000000	0.000000	0.000000	0.000000	0.000000	0.000000	⋯	0.000000	0.000000
4	36	0.000000	0.000000	0.000000	0.000000	0.000000	0.000000	⋯	0.000000	0.067499
5	54	0.000000	0.000000	0.000000	0.000000	0.000000	0.000000	⋯	0.000000	0.000000
6	48	0.000000	0.000000	0.000000	0.000000	0.000000	0.043116	⋯	0.000000	0.000000
7	19	0.000000	0.000000	0.046204	0.000000	0.000000	0.000000	⋯	0.000000	0.035123
8	22	0.000000	0.000000	0.000000	0.000000	0.03884	0.046686	⋯	0.000000	0.000000
9	26	0.000000	0.000000	0.000000	0.000000	0.09395	0.000000	⋯	0.099759	0.000000
⋮	⋮	⋮	⋮	⋮	⋮	⋮	⋮	⋱	⋮	⋮
27462	48	0.040284	0.000000	0.000000	0.000000	0.019002	0.040284	⋯	0.000000	0.01959
27463	76	0.000000	0.000000	0.000000	0.000000	0.000000	0.000000	⋯	0.000000	0.098733
Mean.	-	0.012285								
Std.	-	0.037705								

**Fig 3:**
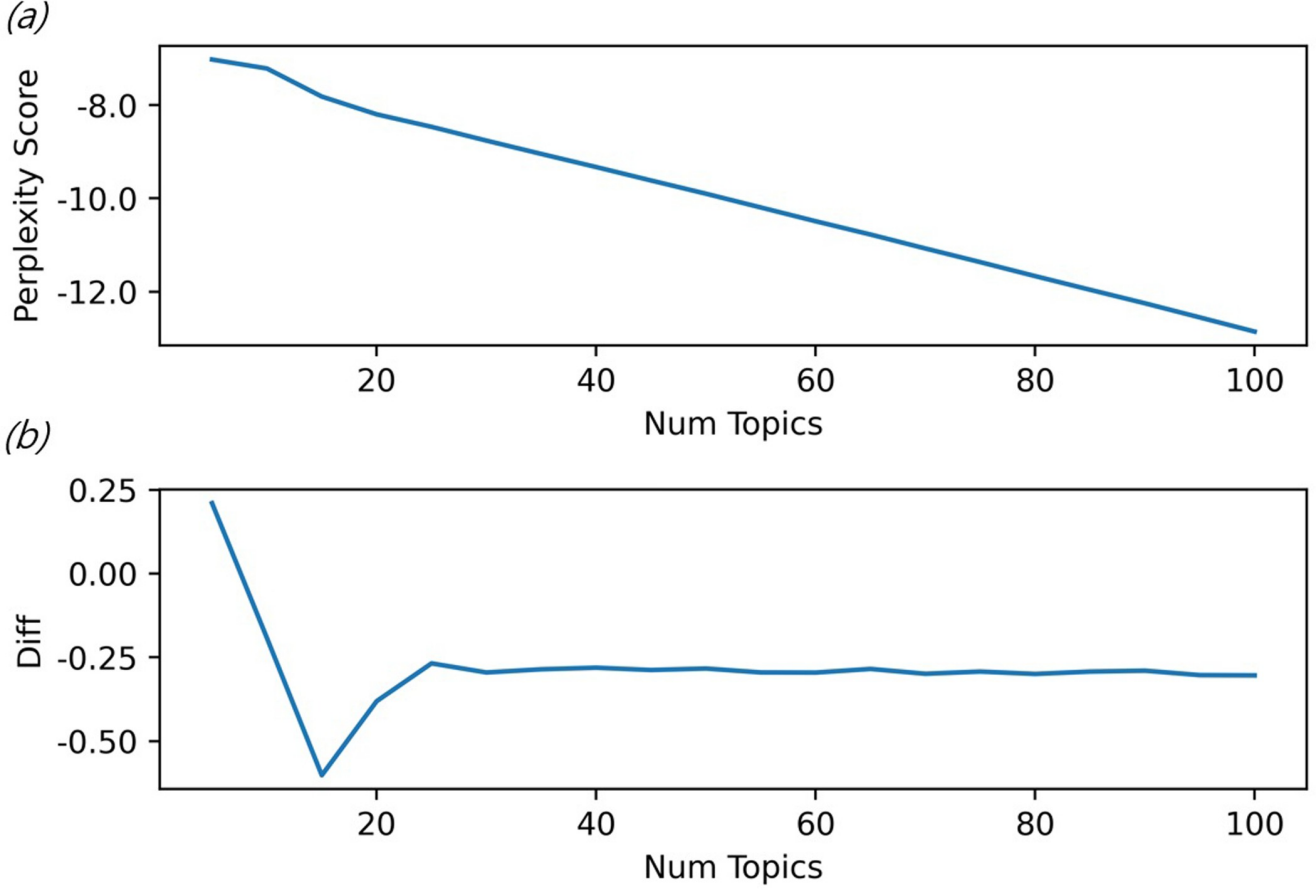
Perplexity score results for selecting optimal topics. [a: Perplexity score by topic. b: Perplexity score increase/decrease rate by topic].

**Fig 4:**
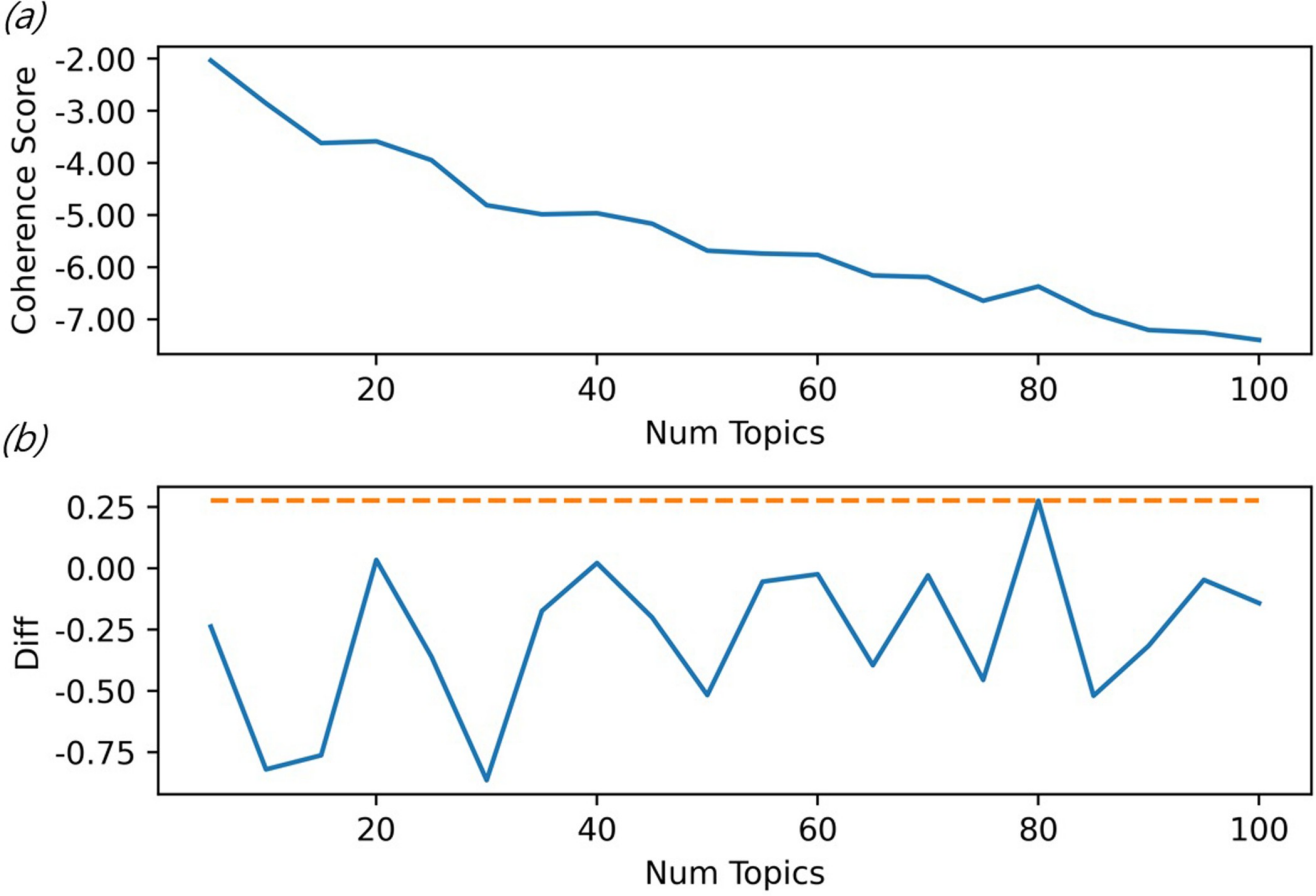
Coherence score results for selecting optimal topics. [a: Coherence score by topic. b: Coherence score increase/decrease rate by topic].

The number of topics was fixed to calculate the optimal passes, and the perplexity per pass was calculated. As displayed in Fig 3, the perplexity score continuously decreases as the number of topics increases. However, Fig 4 confirms that coherence value increased when the number of topics reached 80. The semantic consistency increases when there are 80 topics, and accordingly, 80 was selected as the number of topics. Table 5 presents the calculation results of variable importance using the random forest technique. The importance was the highest in Topic 54, followed by Topic 1 and Topic 15.

**Table 5:** Feature importance results using random forest.

Rank	Topic	Score	Rank	Topic	Score	Rank	Topic	Score	Rank	Topic	Score
1	Topic 54	0.02756	11	Topic 75	0.019603	21	Topic 44	0.015116	31	Topic 41	0.013389
2	Topic 1	0.024331	12	Topic 24	0.018978	22	Topic 78	0.015109	32	Topic 64	0.013241
3	Topic 15	0.024219	13	Topic 39	0.018893	23	Topic 4	0.015069	33	Topic 35	0.012726
4	Topic 57	0.023827	14	Topic 38	0.018621	24	Topic 32	0.014949	34	Topic 6	0.01271
5	Topic 7	0.023359	15	Topic 19	0.01819	25	Topic 49	0.0149	35	Topic 55	0.012489
6	Topic 70	0.020392	16	Topic 3	0.018044	26	Topic 27	0.0143	36	Topic 30	0.012313
7	Topic 65	0.020194	17	Topic 52	0.017669	27	Topic 40	0.014167	37	Topic 47	0.011906
8	Topic 37	0.01982	18	Topic 71	0.015812	28	Topic 14	0.013983	38	Topic 51	0.011846
9	Topic 36	0.019755	19	Topic 58	0.015501	29	Topic 8	0.013901	39	Topic 74	0.011742
10	Topic 68	0.019631	20	Topic 12	0.015207	30	Topic 79	0.013881	40	Topic 10	0.011513

### Patent market value extraction

**Fig 5:**
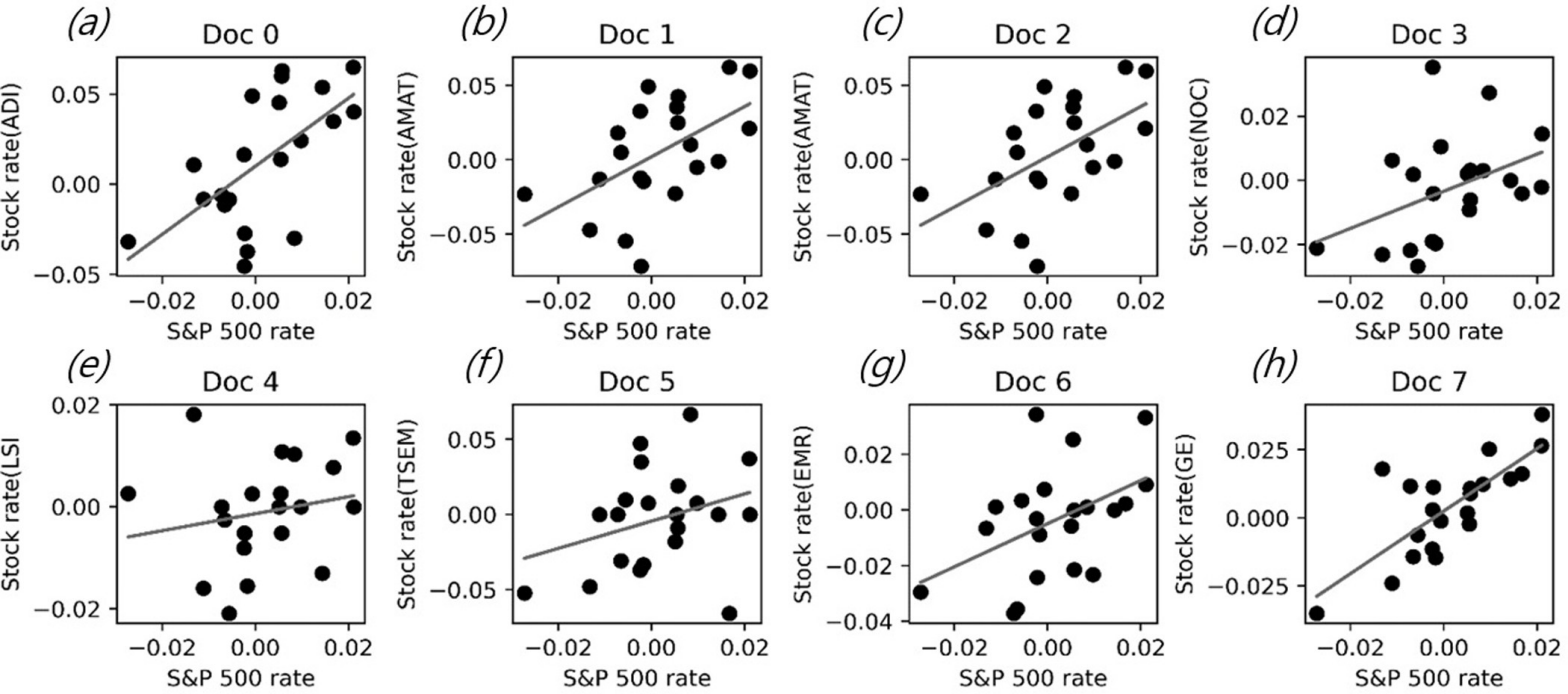
Regression results for the stock return and index return for the first eight patents. [stock name compared to S&P 500 index—a: analog devices, b-c: applied materials, d: northrop grumman, e: life storage, f: tower semiconductor, g: Emerson Electric, h: GE].

To calculate the marketable value of a patent using the event study methodology, the corporation stock price return and S&P 500 return data for the past 30 days were collected based on the registration date of each patent document. A regression analysis was performed by setting the S&P 500 return as an independent variable and the corporation stock price return as a dependent variable. Based on the coefficient information derived from the regression analysis, the abnormal return value of the patent registration date was calculated. Table 6 is a sample of abnormal return calculation results. Fig 5 is the regression analysis result for calculating the abnormal return of the first eight patents among all patents. Each point is the stock return and S&P 500 return value by date, and the solid line is the regression line for each point. Such a regression analysis was performed for each patent document and was performed a total of 27,464 times. Table 7 presents the basic statistics on the calculation results of the abnormal returns for each subtechnical field for all patent documents.

**Table 6:** Sample of abnormal return calculation results.

Document No: 27402		
Division	Stock rate	Index rate
2020-03-16	-16.19%	-12.77%
2020-03-17	6.57%	5.82%
2020-03-18	-11.81%	-5.32%
2020-03-19	6.04%	0.47%
…	…	…
2020-04-13	2.37%	-1.02%
2020-04-14	5.64%	3.01%
Division	Results	
Regression	*R*_27402, *t*_ = -0.00148 + 1.10405 × *RM*_*t*_	
Normal return	-0.00148 + 1.10405 × 3.01% = 3.18%	
Abnormal return	5.64% - 3.18% = 2.46%	

**Table 7:** Basic statistics on the calculation results of abnormal returns for each subtechnical field of all patent document.

IPC		Abnormal return				
		Numbers	Mean	Std.	Max	Min
H01	Basic electric elements	8,974	-0.00101	0.028359	0.660794	-0.29761
H02	Generation, conversion, or distribution of electric power	1,395	-0.00024	0.022233	0.192215	-0.35347
H03	Basic electronic circuitry	2,872	-0.0014	0.02338	0.199513	-0.27639
H04	Electric communication technique	12,934	0.000155	0.016913	0.232953	-0.22644
H05	Electric techniques not otherwise provided for	1,273	-0.00074	0.023346	0.15742	-0.23387
Others		17	-0.01226	0.012531	0.011139	-0.04862
Total		27,464	-0.00045	0.022487	0.660793	-0.35346

### Base learner and ensemble model setting

In this study, tuning was conducted only for the base learner, and hyperparameter tuning was conducted based on the grid search to minimize the adaptation of the validation data in the ensemble model. The variables subject to base learner tuning were limited to the number of trees (random forest), epochs (MLP and CNN), and nodes (MLP and CNN) and were tuned. The training set was divided into seven folds to calculate the prediction value of the base learner for meta learner learning. In addition, each fold was subdivided into sub-training and sub-validation sets, and the optimal hyperparameter of the base learner was calculated for each fold. Table 8 presents the hyperparameter-tuning results of the base learner based on the random forest technique. For Fold 1, as the error rate decreased with an increasing number of trees but increased when the number of trees exceeded 1400, the optimal number of trees was selected as 1400. Tables 9 and 10 present the hyperparameter tuning results of the base learner based on MLP and CNN, respectively. For MLP and CNN, the numbers of nodes and epochs needed to be determined, after which hyperparameter tuning was conducted by building a grid with two variables. First, the optimal epoch was calculated for each node, and then the node whose error rate was the lowest was selected as the optimal node. For Fold 1 of MLP, the lowest error rate was achieved when the number of nodes was 300. The CNN was also constructed using the same process, and for Fold 1, the optimal number of nodes was set to 100.

**Table 8:** Hyperparameter tuning result by fold: Random forest.

Base learner 1		Number of trees										Optimal number of trees
		100	200	400	600	800	1000	1200	1400	1600	1800	
RF	fold1	1.761%	1.745%	1.737%	1.732%	1.731%	1.731%	1.730%	1.730%	1.731%	1.731%	1400
	fold2	3.436%	3.432%	3.425%	3.423%	3.422%	3.419%	3.418%	3.418%	3.417%	3.416%	1800
	fold3	3.931%	3.928%	3.922%	3.920%	3.920%	3.918%	3.918%	3.918%	3.917%	3.916%	1800
	fold4	2.932%	2.926%	2.921%	2.921%	2.919%	2.919%	2.918%	2.918%	2.918%	2.918%	1200
	fold5	1.384%	1.375%	1.368%	1.367%	1.367%	1.365%	1.365%	1.365%	1.364%	1.364%	1800
	fold6	1.682%	1.666%	1.658%	1.652%	1.650%	1.649%	1.648%	1.647%	1.647%	1.647%	1600
	fold7	1.582%	1.572%	1.567%	1.567%	1.568%	1.568%	1.568%	1.569%	1.569%	1.568%	600

**Table 9:** Hyperparameter tuning result by fold: MLP.

Base learner 2			Epoch					Optimal Epoch	Optimal Node
Division		Number of nodes	100	200	300	400	500		
MLP	fold1	50	2.384%	2.700%	2.810%	2.758%	2.847%	100	300
		100	2.509%	2.667%	2.761%	2.646%	2.680%	100	
		150	2.333%	2.364%	2.353%	2.445%	2.392%	100	
		200	2.167%	2.256%	2.198%	2.255%	2.190%	100	
		300	2.141%	2.071%	2.101%	2.089%	2.027%	200	
	fold2	50	4.251%	4.391%	4.583%	4.537%	4.540%	100	50
		100	4.723%	4.815%	4.818%	4.796%	4.868%	100	
		150	4.851%	4.847%	4.906%	4.880%	4.911%	200	
		200	4.889%	4.875%	4.872%	4.916%	4.897%	300	
		300	4.967%	4.898%	4.848%	4.856%	4.922%	300	
	fold3	50	4.343%	4.476%	4.405%	4.396%	4.457%	100	50
		100	4.592%	4.537%	4.545%	4.609%	4.619%	200	
		150	4.591%	4.633%	4.568%	4.650%	4.589%	100	
		200	4.658%	4.656%	4.667%	4.674%	4.582%	200	
		300	4.517%	4.691%	4.565%	4.591%	4.588%	100	
	fold4	50	3.094%	3.194%	3.102%	3.122%	3.159%	100	50
		100	3.165%	3.151%	3.179%	3.150%	3.168%	200	
		150	3.153%	3.143%	3.143%	3.152%	3.175%	300	
		200	3.167%	3.145%	3.157%	3.159%	3.175%	200	
		300	3.177%	3.177%	3.138%	3.150%	3.170%	100	
	fold5	50	1.850%	1.872%	1.894%	1.854%	1.900%	100	50
		100	2.016%	2.040%	2.064%	2.050%	2.077%	100	
		150	2.042%	2.067%	2.076%	2.063%	2.057%	100	
		200	2.096%	2.074%	2.104%	2.097%	2.073%	200	
		300	2.066%	2.076%	2.087%	2.110%	2.078%	100	
	fold6	50	2.025%	2.020%	2.083%	2.055%	2.086%	200	50
		100	2.103%	2.129%	2.158%	2.131%	2.172%	100	
		150	2.180%	2.203%	2.175%	2.195%	2.192%	100	
		200	2.219%	2.152%	2.174%	2.179%	2.181%	200	
		300	2.168%	2.174%	2.191%	2.181%	2.184%	100	
	fold7	50	1.989%	2.140%	2.151%	2.166%	2.132%	100	50
		100	2.215%	2.269%	2.244%	2.284%	2.248%	100	
		150	2.249%	2.270%	2.276%	2.247%	2.306%	100	
		200	2.359%	2.244%	2.307%	2.270%	2.287%	200	
		300	2.294%	2.342%	2.311%	2.282%	2.242%	100	

**Table 10:** Hyperparameter tuning result by fold: CNN.

Base learner			Epoch					Optimal Epoch	Optimal Node
Division		Number of nodes	100	200	300	400	500		
CNN	fold1	50	1.716%	1.745%	1.745%	1.723%	1.716%	100	100
		100	1.705%	1.725%	1.752%	1.733%	1.715%	100	
		150	1.714%	1.717%	1.751%	1.730%	1.711%	100	
		200	1.711%	1.714%	1.743%	1.721%	1.713%	100	
		300	1.711%	1.718%	1.747%	1.720%	1.714%	100	
	fold2	50	3.306%	3.327%	3.351%	3.315%	3.358%	100	300
		100	3.308%	3.319%	3.339%	3.358%	3.379%	100	
		150	3.293%	3.308%	3.355%	3.334%	3.342%	100	
		200	3.304%	3.306%	3.333%	3.337%	3.347%	100	
		300	3.303%	3.284%	3.359%	3.340%	3.337%	200	
	fold3	50	3.834%	3.839%	3.846%	3.839%	3.845%	100	150
		100	3.834%	3.843%	3.843%	3.847%	3.848%	100	
		150	3.834%	3.830%	3.844%	3.844%	3.850%	200	
		200	3.831%	3.834%	3.840%	3.846%	3.847%	100	
		300	3.833%	3.832%	3.844%	3.841%	3.843%	200	
	fold4	50	2.870%	2.872%	2.875%	2.875%	2.886%	100	300
		100	2.870%	2.881%	2.866%	2.864%	2.888%	100	
		150	2.865%	2.881%	2.867%	2.870%	2.884%	100	
		200	2.866%	2.885%	2.867%	2.857%	2.891%	100	
		300	2.861%	2.873%	2.868%	2.858%	2.886%	100	
	fold5	50	1.343%	1.345%	1.367%	1.380%	1.374%	100	200
		100	1.342%	1.355%	1.356%	1.388%	1.374%	100	
		150	1.328%	1.337%	1.358%	1.375%	1.364%	100	
		200	1.327%	1.344%	1.383%	1.383%	1.372%	100	
		300	1.338%	1.341%	1.355%	1.372%	1.331%	100	
	fold6	50	1.630%	1.630%	1.650%	1.645%	1.642%	100	200
		100	1.628%	1.626%	1.647%	1.635%	1.643%	200	
		150	1.631%	1.627%	1.647%	1.631%	1.642%	200	
		200	1.627%	1.624%	1.643%	1.638%	1.634%	200	
		300	1.635%	1.636%	1.643%	1.631%	1.631%	200	
	fold7	50	1.559%	1.584%	1.576%	1.578%	1.580%	100	100
		100	1.553%	1.577%	1.578%	1.577%	1.575%	100	
		150	1.560%	1.567%	1.589%	1.580%	1.564%	100	
		200	1.558%	1.568%	1.587%	1.571%	1.565%	100	
		300	1.554%	1.556%	1.577%	1.568%	1.566%	100	

Table 11 summarizes the hyperparameter tuning results of the meta learner (SVR) with the input of the prediction value of the base learner (random forest, MLP, and CNN). A dataset was built by gathering prediction values of each fold, which was then divided into a sub-training set and sub-validation set to perform a grid search. For SVR, the regularization parameter was the tuning target, and the optimal value was calculated as 1.

**Table 11:** Hyperparameter tuning result of meta learner (SVR).

Meta learner		C (Regularization parameter)										Optimal C
		1	2	3	4	5	10	25	50	100	200	
SVR	MAE	0.940%	0.951%	0.953%	0.950%	0.955%	0.962%	0.966%	0.975%	0.993%	0.992%	1
	MSE	0.022%	0.022%	0.023%	0.022%	0.023%	0.023%	0.023%	0.023%	0.023%	0.023%	
	RMSE	1.489%	1.498%	1.500%	1.498%	1.502%	1.507%	1.510%	1.518%	1.532%	1.532%	

Table 12 summarizes the hyperparameters of the base learner and meta learner used in the ensemble model. MSE was used as a loss function in the random forest, MLP, and CNN models. Gini was employed as a criterion of the random forest model, and Adam was used as the optimizer of MLP and CNN. For the CNN, the filter, kernel, and pool sizes were set as 32, 2, and 2, respectively. For SVR, which was used as the meta learner, the kernel used was linear and the cache size was set as 200.

**Table 12:** Hyperparameter setting results of ensemble model.

Divisions	Models	Hyperparameters		Values
Base learner	Random Forest	Number of trees	Fold1	1400
			Fold2	1800
			Fold3	1800
			Fold4	1200
			Fold5	1800
			Fold6	1600
			Fold7	600
		Loss function		MSE
		Criterion		Gini
		Random state		1
	MLP	Number of nodes	Fold1	300
			Fold2	50
			Fold3	50
			Fold4	50
			Fold5	50
			Fold6	50
			Fold7	50
		Epoch	Fold1	200
			Fold2	100
			Fold3	100
			Fold4	100
			Fold5	100
			Fold6	200
			Fold7	100
		Number of hidden layers		2
		Loss function		MSE
		Optimizer		Adam
	CNN	Number of nodes(Epoch)	Fold1	100
			Fold2	300
			Fold3	150
			Fold4	300
			Fold5	200
			Fold6	200
			Fold7	100
		Epoch	Fold1	100
			Fold2	200
			Fold3	200
			Fold4	100
			Fold5	100
			Fold6	200
			Fold7	100
		Dimension		1
		Filter size		32
		Kernel size		2
		Pool size		2
		Loss function		MSE
		Optimizer		Adam
Meta learner (Stacking Ensemble Model)	SVR	Kernel		Linear
		C (Regularization parameter)		1
		Epsilon		0.1
		Cache size		200
		Max iteration		No limit

**Fig 6:**
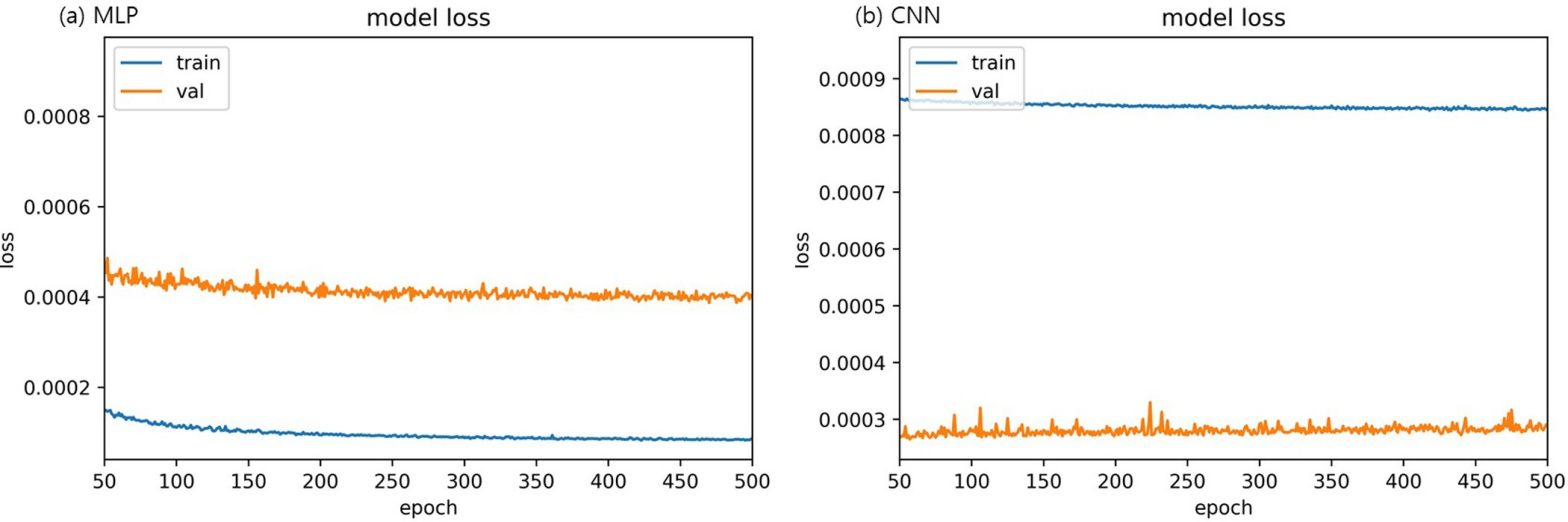
Changes in the loss rate according to an increase in epochs [a: prediction error of MLP model. b: prediction error of CNN model].

A control group is needed to determine the predictive power of the ensemble model. As a control group, linear regression, random forest, MLP, and CNN were used, and hyperparameter tuning of random forest, MLP, and CNN, which required hyperparameter tuning, was performed. Table 13 presents the hyperparameter tuning results of the control group model. For the control group, because no fold needed to be built, a training set was used in learning. The optimal number of trees in the random forest, which was a control group, was 400. The optimal numbers of nodes in MLP and CNN were calculated as 300 and 50, respectively. Fig 6 shows the comparison result of the optimal epoch calculation process between MLP and CNN control group models using training and validation sets.

**Table 13:** Hyperparameter tuning result of control group.

Division		Results									
RF	Number of trees	100	200	400	600	800	1000	1200	1400	1600	1800
	MAE	1.0090%	0.9934%	0.9826%	0.9807%	0.9802%	0.980%	0.979%	0.980%	0.980%	0.979%
	MSE	0.0287%	0.0282%	0.0279%	0.0279%	0.0279%	0.028%	0.028%	0.028%	0.028%	0.028%
	RMSE	1.6930%	1.6802%	1.6698%	1.6705%	1.6707%	1.670%	1.668%	1.668%	1.668%	1.666%
	Optimal number of trees	400									
MLP	Nodes	50					100				
	Epoch	100	200	300	400	500	100	200	300	400	500
	MAE	1.9217%	2.1197%	2.2757%	2.2985%	2.3921%	1.9050%	1.9327%	1.9808%	1.9231%	2.0401%
	MSE	0.0764%	0.0954%	0.1021%	0.1046%	0.1154%	0.0701%	0.0713%	0.0759%	0.0710%	0.0769%
	RMSE	2.7648%	3.0881%	3.1954%	3.2348%	3.3969%	2.6468%	2.6697%	2.7545%	2.6648%	2.7725%
	Nodes	150					200				
	Epoch	100	200	300	400	500	100	200	300	400	500
	MAE	1.6414%	1.6369%	1.6387%	1.5884%	1.6139%	1.5523%	1.4678%	1.4754%	1.4243%	1.4263%
	MSE	0.0540%	0.0542%	0.0542%	0.0517%	0.0523%	0.0507%	0.0464%	0.0464%	0.0444%	0.0438%
	RMSE	2.3230%	2.3286%	2.3274%	2.2730%	2.2862%	2.2510%	2.1543%	2.1538%	2.1060%	2.0932%
	Nodes	300									
	Epoch	100	200	300	400	500					
	MAE	1.3848%	1.3422%	1.3527%	1.3313%	1.3139%					
	MSE	0.0423%	0.0407%	0.0406%	0.0398%	0.0392%					
	RMSE	2.0565%	2.0167%	2.0144%	1.9958%	1.9802%					
	Optimal Node	300									
	Optimal Epoch	500									
CNN	Nodes	50					100				
	Epoch	100	200	300	400	500	100	200	300	400	500
	MAE	1.0314%	0.9701%	0.9948%	1.0135%	1.0439%	1.0332%	0.9771%	0.9894%	1.0157%	1.0376%
	MSE	0.0284%	0.0271%	0.0278%	0.0283%	0.0289%	0.0284%	0.0273%	0.0276%	0.0282%	0.0286%
	RMSE	1.6856%	1.6460%	1.6668%	1.6837%	1.7001%	1.6851%	1.6516%	1.6607%	1.6799%	1.6913%
	Nodes	150					200				
	Epoch	100	200	300	400	500	100	200	300	400	500
	MAE	1.0299%	0.9775%	0.9876%	0.9933%	1.0355%	1.0196%	0.9920%	0.9939%	0.9999%	1.0449%
	MSE	0.0283%	0.0272%	0.0276%	0.0277%	0.0287%	0.0281%	0.0276%	0.0276%	0.0280%	0.0287%
	RMSE	1.6821%	1.6503%	1.6618%	1.6648%	1.6940%	1.6773%	1.6611%	1.6627%	1.6728%	1.6940%
	Nodes	300									
	Epoch	100	200	300	400	500					
	MAE	1.0221%	0.9912%	0.9828%	0.9887%	1.0206%					
	MSE	0.0282%	0.0275%	0.0273%	0.0277%	0.0282%					
	RMSE	1.6791%	1.6583%	1.6536%	1.6636%	1.6791%					
	Optimal Node	50									
	Optimal Epoch	200									

## Experiment results

**Fig 7:**
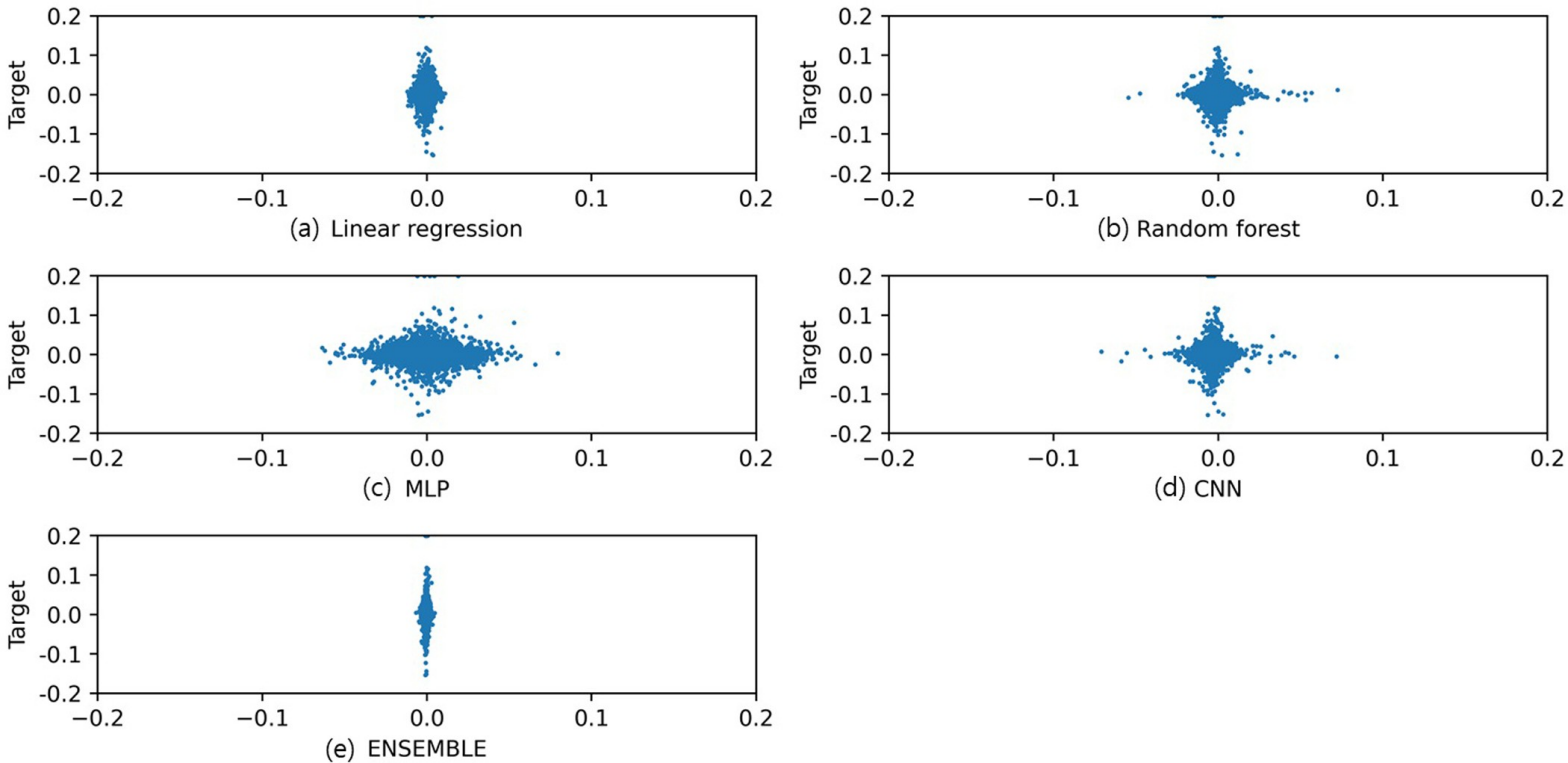
Prediction comparison (horizontal axis: Prediction value, vertical axis: Target value). [a: Linear regression model predicted value and target value scatterplot, b: Random forest model predicted value and target value scatterplot, c: MLP model predicted value and target value scatterplot, d: CNN model predicted value and target value scatterplot, e: Ensemble model predicted value and target value scatterplot].

The RF, MLP, and CNN models were separately trained with equivalent training data to compare the predictive power of the SVR-based stacking ensemble model. Then, the predictive power of each model was verified on the testing set. Fig 7 presents the correspondence between the predicted market value and actual target value of each model. The upper left is the RF model, and the upper right is MLP. The lower left is the CNN, and the lower right is the prediction results of the stacking ensemble model. In each figure, the horizontal axis represents the predicted model value, and the vertical axis represents the actual target value. The precision of the MLP was relatively inferior to that of the other models, and the precision of the stacking ensemble model was the highest.

Table 14 illustrates the error results for each model. As a result of the error analysis, the stacking ensemble model exhibited the lowest error in all error measures. Based on the RMSE, the predictive power of the stacking ensemble model was 1.013 times higher than that of the Linear regression model, 1.033 times higher than that of the Random forest model, 1.194 times higher than that of the MLP model, and 1.062 times higher than that of the CNN model.

**Table 14:** Prediction error results for each model.

Division	Model 1	Model 2	Model 3	Model 4	Model 5
	Linear Regression	Random Forest	MLP	CNN	Stacking Ensemble(SVR)
MAE	0.010543	0.010885	0.013571	0.011505	0.010326
MSE	0.00027	0.000281	0.000375	0.000297	0.000263
RMSE	0.01644	0.016775	0.019376	0.017238	0.016232

Table 15 lists the Anderson–Darling normality test results for each pair. As a result of testing, all pairs exhibited non-normality. Table 16 presents the Wilcoxon rank-sum test results of each pair. The predicted value of the stacking ensemble and linear regression models (Pair 1) exhibited a statistically significant difference, and Pairs 2, 3 and 4 also demonstrated statistically significant differences. Therefore, the predictive power of the ensemble model was superior to that of the other single models, and the difference in predictive power from the single model was statistically significant.

**Table 15:** Results of normality test (Anderson-Darling normality test).

Division	Pair composition		Anderson-Darling normality test		
	Set 1	Set 2	*A*	*p*-value	Results
Pair 1	Stacking Ensemble	Linear Regression	6.1499	4.15e-15	Non-normal
Pair 2	Stacking Ensemble	Random Forest	201.63	< 2.2e-16	Non-normal
Pair 3	Stacking Ensemble	MLP	73.735	< 2.2e-16	Non-normal
Pair 4	Stacking Ensemble	CNN	219.06	< 2.2e-16	Non-normal

**Table 16:** Results of the Wilcoxon rank-sum test with continuity correction.

Division	Pair composition		Wilcoxon rank-sum test		
	Set 1	Set 2	*W*	*p*-value	Results
Pair 1	Stacking Ensemble	Linear Regression	35590629	6.5e-08	difference
Pair 1	Stacking Ensemble	Random Forest	33190363	0.01401	difference
Pair 2	Stacking Ensemble	MLP	36527474	< 2.2e-16	difference
Pair 3	Stacking Ensemble	CNN	8991146	< 2.2e-16	difference

## Conclusion

In this study, the patent text analysis methodology for patent valuation was combined with an event study. Among the event study methodologies, an analysis was performed based on a market model, and the calculated abnormal return was used as the market value of each patent. In the patent text analysis, LDA-based topic modeling was used to capture the semantic characteristics of the patent. Perplexity and coherence analyses were used to calculate the optimal number of topics. The coherence analysis found the optimal number of topics to be 80, which indicates that semantic consistency was highest when all patent documents were divided into 80 categories.

In addition, the marketable value of the patent was estimated using the SVR-based ensemble model. The RF, MLP, and CNN models were used as the base learners of the ensemble model. Considering the ease of interpretation, the SVR was used as the ensemble learning algorithm. The RF, MLP, and CNN were trained separately using equivalent training data to determine the predictive power of the ensemble model. After comparing the predictive power of each model using the MAE, MSE, and RMSE values, the predictive power of the ensemble model was the highest. The base learners with high predictive power for each fold were different. However, the ensemble model trained on the base learners’ predicted values exceeded the predictive power of each of the single models, which indicates that the predictive power of the ensemble model is more stable than that of the single model.

To check whether the predicted values of the stacking ensemble model and the predicted values of the individual models exhibited statistically significant differences, the Anderson–Darling normality and Wilcoxon rank-sum tests were performed. The analysis revealed that the predicted value of the stacking ensemble model exhibited a statistically significant difference from the predicted value of a single model using the RF, MLP and CNN. Therefore, the predictive power of the ensemble model was superior to that of other single models, and the difference in predictive power from the single model was statistically significant.

These models employ the stock price change at the patent registration date to calculate the target variable. However, this approach has the following limitations. First, “information other than patent”, which is not controlled as the benchmark index, may be introduced to the abnormal return term although this approach assumes that the price-earnings ratio is divided into normal and abnormal returns and the abnormal return value at the patent registration date is structured by the patent events. Second, patent development information may be introduced to the market in advance before the patent registration date. In such a case, an increase or decrease in market value according to the patent registration can be reflected in advance, thereby producing an overestimated or underestimated value. Thus, we believe a study on improving the method to extract patent value is needed as follow-up research. If this methodology becomes popular, the keywords of patent specifications may be manipulated so as to ensure a high patent valuation. In such a case, evaluation may be required through multiple methodologies. In addition, the present study used three base learners (random forest, MLP, and CNN) to combine the calculated patent value and text information, and SVR was employed as a meta learner, but various other algorithms may be considered in the future study.

## Supporting information

S1 Data (ZIP)
